# Low socioeconomic status, ethnicity and geographical location confers high risk of significant accidental burns injuries in London

**DOI:** 10.1186/cc13267

**Published:** 2014-03-17

**Authors:** JS Heng, O Clancy, I Jones, J Atkins, J Leon-Villapalos, A Williams, M Hayes, M Vizcaychipi

**Affiliations:** 1 Chelsea & Westminster Hospital, London, UK

## Introduction

The majority of burns injuries are considered accidental, although previous studies have identified demographic factors associated with higher risk of burns such as socioeconomic deprivation [1] and being from ethnic minority groups [2]. This study aims to identify population subgroups in London at high risk of burns injuries requiring admission to a burns centre through geographic mapping and socioeconomic statistics.

## Methods

Records of all paediatric and adult inpatients admitted to the burns centre at Chelsea and Westminster Hospital were retrospectively reviewed for age, ethnic group and deprivation score of residence, as measured by the English Index of Multiple Deprivation 2010. Corresponding population data for London were obtained.

## Results

In total, 2,195 patients from London were admitted between January 2009 and August 2013, with 1,963 (89.4%) classified as having accidental injuries. A total 1,725 (87.8%) of accidental burn injuries occurred in the patients' own homes. Patients from ethnic minorities have the highest rate of burn injury at 7.1 per 100,000 population per annum (*P *< 0.0001). Patients below the median for socioeconomic deprivation in London are more likely to suffer burn injuries (*P *< 0.0001). Patients from the most deprived quartile are more likely to suffer burns injuries of >10% TBSA (*P *= 0.04), and have a trend towards higher rates of ICU admission (*P *= 0.144). Domestic accidental burns were mapped to their respective administrative wards, and the rate of burns per 100,0 was calculated and divided into quintiles. The areas with the top quintile of burn injury rate, of up to 18.8 per 100,000 population per year, were almost four times the national average.

## Conclusion

Ethnicity, socioeconomic deprivation and geographical location appear to be risk factors for burn injuries. Identifying such groups may allow the development of targeted preventative strategies.
